# Sparse learning and stability selection for predicting MCI to AD conversion using baseline ADNI data

**DOI:** 10.1186/1471-2377-12-46

**Published:** 2012-06-25

**Authors:** Jieping Ye, Michael Farnum, Eric Yang, Rudi Verbeeck, Victor Lobanov, Nandini Raghavan, Gerald Novak, Allitia DiBernardo, Vaibhav A Narayan

**Affiliations:** 1 Center for Evolutionary Medicine and Informatics, The Biodesign Institute, Arizona, State University, Tempe, AZ, USA; 2 Johnson & Johnson Pharmaceutical Research & Development, LLC, Titusville, NJ, USA

## Abstract

**Background:**

Patients with Mild Cognitive Impairment (MCI) are at high risk of progression to Alzheimer’s dementia. Identifying MCI individuals with high likelihood of conversion to dementia and the associated biosignatures has recently received increasing attention in AD research. Different biosignatures for AD (neuroimaging, demographic, genetic and cognitive measures) may contain complementary information for diagnosis and prognosis of AD.

**Methods:**

We have conducted a comprehensive study using a large number of samples from the Alzheimer’s Disease Neuroimaging Initiative (ADNI) to test the power of integrating various baseline data for predicting the conversion from MCI to probable AD and identifying a small subset of biosignatures for the prediction and assess the relative importance of different modalities in predicting MCI to AD conversion. We have employed sparse logistic regression with stability selection for the integration and selection of potential predictors. Our study differs from many of the other ones in three important respects: (1) we use a large cohort of MCI samples that are unbiased with respect to age or education status between case and controls (2) we integrate and test various types of baseline data available in ADNI including MRI, demographic, genetic and cognitive measures and (3) we apply sparse logistic regression with stability selection to ADNI data for robust feature selection.

**Results:**

We have used 319 MCI subjects from ADNI that had MRI measurements at the baseline and passed quality control, including 177 MCI Non-converters and 142 MCI Converters. Conversion was considered over the course of a 4-year follow-up period. A combination of 15 features (predictors) including those from MRI scans, APOE genotyping, and cognitive measures achieves the best prediction with an AUC score of 0.8587.

**Conclusions:**

Our results demonstrate the power of integrating various baseline data for prediction of the conversion from MCI to probable AD. Our results also demonstrate the effectiveness of stability selection for feature selection in the context of sparse logistic regression.

## Background

Alzheimer’s disease (AD) is the most common type of dementia, accounting for 60–80% of age-related dementia cases [1]. AD currently affects about 5.3 million people in the US, with a significant increase predicted in the near future if no disease-altering therapeutics are developed [1]. In AD patients, neurons and their connections are progressively destroyed, leading to loss of cognitive function and ultimately death. As therapeutic intervention is most likely to be beneficial in the early stage of the disease, identification of a biosignature that enables an earlier and more accurate diagnosis of AD is an important goal. Mild Cognitive Impairment (MCI), a transitional stage between normal aging and the development of dementia, has been introduced to account for the intermediate cognitive state where patients are impaired on one or more standardized cognitive tests but do not meet the criteria for clinical diagnosis of dementia [2]. The American Academy of Neurology has recognized MCI as an important clinical group to be identified and monitored [3]. Patients with MCI are at high risk of progression to dementia; it is estimated that 10–15% of these patients progress to AD annually. MCI has thus attracted increasing attention, because it offers an opportunity to target the disease process early. More recently, MCI has been further classified according to the presence or absence of a primary memory deficit (amnestic and nonamnestic MCI, respectively), either in relative isolation (single domain) or accompanied by other types of cognitive deficits (multiple domain). As the amnestic form of MCI, single or multiple domain, has the greatest risk of progression to dementia, it has been a primary focus of interest in aging studies. There is thus an urgent need to address two major research questions: (1) how can we identify MCI individuals with high likelihood of progression to dementia (2) what is the biosignature most predictive of the conversion from MCI to AD. Brain atrophy measured by MRI scans, positron emission tomography (PET) including imaging of amyloid burden, and CSF measurements including Aβ_42_ and total tau (t-tau) have been the prime candidate biosignatures for diagnosis and tracking disease progression.

Neuroimaging has been shown to be a powerful tool for the ex ploration of disease progression and therapeutic efficacy in AD and MCI. Neuroimaging research offers great potential to identify features that can identify individuals early in the course of dementing illness; several candidate neuroimaging biosignatures have been examined in recent cross-sectional and longitudinal neuroimaging studies [4,5]. Realizing the importance of neuroimaging, NIH in 2003 funded the Alzheimer’s Disease Neuroimaging Initiative (ADNI). All subjects in ADNI undergo 1.5T or 3T structural Magnetic Resonance Imaging (MRI) scans. Half of the subjects undergo Positron Emission Tomography (PET) scans. While FDG-PET scans may show a high sensitivity or specificity for the early detection of AD, the validation of structural MRI markers is the core project in ADNI due to its greater availability, faster data acquisition, and lower cost. Structural MRI, in particular, has great potential in enabling earlier clinical diagnosis and predicting disease progression. Previous studies have demonstrated that the hippocampus and the entorhinal cortex of MCI patients are typically smaller than those measured in normal controls, and are predictive of future conversion to AD [4]. As the specificity of the prediction is still low [5], current work continues to examine additional regions and pattern changes for more accurate prediction.

Besides brain atrophy measured by MRI scans, CSF measurements including total tau (t-tau), phosphorylated tau (p-tau), and Aβ_42_ were identified as being among the most promising and informative AD biosignatures. Increased CSF concentrations of t-tau and p-tau and decreased concentrations of Aβ_42_ are found in MCI and AD, and their combination is considered to be characteristic of AD. However, there is considerable variability of published opinion on the utility of CSF measurements for predicting conversion from MCI to AD [6-8]. This may be attributable to the small number of subjects used in many of the previous studies and the variability in their measurement methodology.

In addition to MRI and CSF measurements, there are various clinical/cognitive assessment scores from the ADNI data set that are potentially useful for the prediction of MCI-to-AD conversion, including Mini Mental State Examination (MMSE), Clinical Dementia Rating Sum of Boxes (CDR-SB), Alzheimer’s Disease Assessment Scale-cognitive subscale (ADAS-cog), Logical Memory immediate (LIMM) and delayed (DELL) paragraph recall, Activities of Daily Living Score (from the Functional Activities Questionnaire, FAQ), and Trail Making Tests: Part A (TRAA) and Part B (TRAB). Clinical/cognitive assessments offer potential advantages over imaging or CSF biomarkers since the use of imaging and CSF biomarkers could severely limit the number of participants screened for a study. Although MRI, CSF, and clinical/cognitive assessments have been extensively studied in the past, few reports have compared and combined various measurements from MCI subjects. In this study, we use a large number of samples from ADNI to test:

(1) the ability of various baseline data (MRI, demographic, genetic and cognitive measures) for predicting the conversion from MCI to probable AD

(2) the power of integrating various baseline data in order to identify a biosignature (small subset of predictive biomarkers) for prediction of the conversion from MCI to probable AD and

(3) the use of CSF biomarkers for predicting the conversion from MCI to probable AD and the potential of increasing predictive accuracy by combining CSF biomarkers with other measurements.

The main technical challenge is how to integrate effectively various baseline data for classification (MCI Converts versus MCI Non-converts). A simple approach for data integration is to form a long vector for each sample (subject) by concatenating the features from all baseline data, which is then fed into a classifier such as support vector machines (SVM) [9]. To deal with the high dimension/small sample size problem, feature selection, which selects a small subset of features for improved generalization performance, is commonly applied. Most existing feature selection algorithms such as the *t*-test perform univariate feature ranking [10], and they fail to take the feature correlation into consideration. In this paper, we apply sparse logistic regression for feature selection, which selects a small subset of features using the L_1_- norm regularization [11]. The L_1_-norm regularization is appealing in many applications due to its sparsity-inducing property, convenient convexity, and strong theoretical guarantees [12]. An important issue in the practical application of sparse logistic regression is the selection of an appropriate amount of regularization, known as model selection. Cross validation is commonly used for model selection, however it tends to select more features than needed. In this paper, we employed stability selection, a method recently proposed to address the problem of proper regularization using subsampling/bootstrapping [13].

Our study differs from others in three important respects: (1) we use a large cohort of MCI samples that are unbiased with respect to age or education status between case and controls (2) we integrate and test various types of baseline data available in ADNI including MRI, demographic, genetic and cognitive measures and (3) we apply sparse logistic regression with stability selection to ADNI data for robust feature selection. We have evaluated sparse logistic regression with stability selection on a set of 319 MCI subjects from ADNI, including 177 MCI Non-converters and 142 MCI Converters (the conversion was considered over the course of a 4-year follow-up period). Our experiments show that a combination of 15 features from MRI scans, APOE genotyping, and cognitive measures selected by sparse logistic regression with stability selection achieves an AUC score of 0.8587.

## Methods

### Ethics

In this study we used ADNI data that were previously collected across 50 sites. Study subjects gave written informed consent at the time of enrollment for data collection and completed questionnaires approved by each participating site’s Institutional Review Board (IRB). The complete list of ADNI sites’ IRBs can be found at the link: http://adni.loni.ucla.edu/about/data-statistics/. The authors state that they have obtained approval from the ADNI Data Sharing and Publications Committee for use of the data.

### ADNI participants

The data used in the preparation of this article were obtained from the Alzheimer’s Disease Neuroimaging Initiative (ADNI) database (adni.loni.ucla.edu). Data used for our analyses were accessed on August 8, 2010. The ADNI was launched in 2003 by the National Institute on Aging (NIA), the National Institute of Biomedical Imaging and Bioengineering (NIBIB), the Food and Drug Administration (FDA), private pharmaceutical companies and non-profit organizations, as a $60 million, 5 -year public- private partnership. The primary goal of ADNI has been to test whether serial magnetic resonance imaging (MRI), positron emission tomography (PET), other biological markers, and clinical and neuropsychological assessment can be combined to measure the progression of mild cognitive impairment (MCI) and early Alzheimer’s disease (AD). The identification of sensitive and specific markers of very early AD progression will facilitate the diagnosis of early AD and the development, assessment, and monitoring of new treatments.

The Principal Investigator of this initiative is Michael W. Weiner, MD, VA Medical Center and University of California – San Francisco. ADNI is the result of efforts of many co- investigators from a broad range of academic institutions and private corporations, and subjects have been recruited from over 50 sites across the U.S. and Canada. The initial goal of ADNI was to recruit 800 adults, ages 55 to 90, to participate in the research, approximately 200 cognitively normal older individuals to be followed for 3 years, 400 people with MCI to be followed for 3 years and 200 people with early AD to be followed for 2 years.” For up-to-date information, see http://www.adni-info.org.

### Subject characteristics and schedule of assessments in ADNI

**Figure 1:**
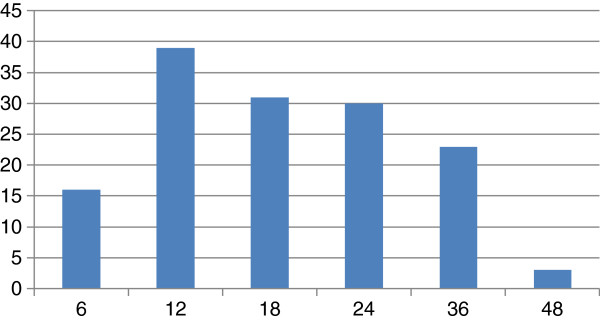
The number of MCI to AD conversions at each time point (6, 12, 18, 24, 36, 48 months).

There were 319 MCI subjects included in this study including 177 MCI Non-converters and 142 MCI Converters. We only used a subset of the MCI subjects from ADNI which had MRI measurements at baseline and passed quality control. The conversion was considered over the course of a 4-year time period. General inclusion/exclusion criteria for MCI subjects are as follows: MMSE scores between 24 and 30 (inclusive; exceptions made on a case-by-case basis), memory complaint, objective memory loss measured by education adjusted scores on Wechsler Memory Scale Logical Memory II, CDR of 0.5, absence of significant levels of impairment in other cognitive domains, essentially preserved activities of daily living, and an absence of dementia. Thus, this corresponds to criteria for amnestic MCI. ADNI eligibility criteria are described at http://www.adni-info.org. MCI individuals at ADNI were assessed by neuroimaging at baseline, 6, 12, 18, 24, 36, 48 months. The number of MCI to AD conversions at each time point (6, 12, 18, 24, 36, 48 months) is summarized in Figure 1.

All participants received 1.5 Tesla (T) structural MRI. The analyses in this study were based on the imaging data from the ADNI database processed by the team at the University of California at San Francisco, which performed cortical reconstruction and volumetric segmentation with the Freesurfer image analysis suite (http://surfer.nmr.mgh.harvard.edu/). The detailed procedure is available at http://adni.loni.ucla.edu/research/imaging-analysis/. A list of 237 MRI features used in this study is provided at the Additional file 1: Supplemental document. More details on ADNI neuroimaging instrumentation and procedures [14] can be found at http://www.loni.ucla.edu. About 50% of MCI subjects (74 MCI Converts, 86 MCI Non-converters) have a complete set of CSF measurements and MRI scans at the baseline. CSF was analyzed at the ADNI Biomarker Core laboratory at the University of Pennsylvania Medical Center.

A number of clinical/cognitive assessment scores were used in this study, including Mini Mental State Examination (MMSE), Clinical Dementia Rating Sum of Boxes (CDR-SB), Alzheimer’s Disease Assessment Scale-cognitive subscale (ADAS-cog), Logical Memory immediate (LIMM) and delayed (DELL) paragraph recall, Activities of Daily Living Score (from the Functional Activities Questionnaire, FAQ), and Trail Making Tests: Part A (TRAA) and Part B (TRAB).

The following 18 lab tests were included in our study: Test RCT1 -Total Bilirubin, Test RCT11-Serum Glucose, Test RCT12-Total Protein, Test RCT13-Albumin, Test RCT14-Creatine Kinase, Test RCT1407-Alkaline Phosphatase, Test RCT1408-Lactate Dehydrogenase (LDH), Test RCT183-Calcium (EDTA), Test RCT19-Triglycerides (GPO), Test RCT20-Cholesterol (High Performance), Test RCT29-Direct Bilirubin, Test RCT3-GGT, Test RCT392-Creatinine (Rate Blanked), Test RCT4-Alanine aminotransferase (ALT), Test RCT5-aspartate aminotransferase, Test RCT6-Urea Nitrogen, Test RCT8-Serum Uric Acid, and Test RCT9-Phosphorus. We report the P-value of various baseline measurements computed by 2 -sample *t*-test. To test the ability of various baseline data for predicting the conversion from MCI to probable AD, we apply support vector machines (SVM) on each type of baseline measurement to build the classifier [9]. SVM finds a maximum margin separating hyperplane between two classes. It leads to a straightforward learning algorithm that can be reduced to a convex optimization problem. We evaluate the prediction performance in terms of the area under the curve (AUC) score [15], commonly used in the literature. Specifically, we report the leave-one-out AUC score, in which we build an SVM model on all but one MCI subject and apply the classification model to predict the left-out MCI subject, and we repeat this procedure for all MCI subjects.

### Biosignature selection via sparse logistic regression with stability selection

We employed sparse logistic regression based on the L_1_ norm regularization for biosignature (feature) selection. Let x∈R^p^ denote a sample of p features, and let y∈{−1,+1} be the associated (binary) class label (y = 1 for MCI Converts and y = −1 for MCI Non-converts). The logistic regression model is given by:

$$\text{Prob}\left(\text{y}|\text{x}\right)=1/\left(1+\text{exp}\left(-\text{y}\left({\text{w}}^{\text{T}}\text{x}+\text{c}\right)\right)\right)$$

where Prob(y|x) is the conditional probability of y, given x, w∈R^p^ is a weight vector, and c∈R is the intercept. The expression w^T^ x + c = 0 defines a hyperplane in the feature space, on which Prob(y|x) = 0.5. The conditional probability Prob(y|x) is larger than 0.5 if w^T^ x + c has the same sign as y, and less than 0.5 otherwise. Suppose that we are given a set of n training data {x_i_,y_i_}, i = 1,2,…,n, where x_i_∈R^p^ denotes the *i*-th sample and y_i_∈{−1,+1} denotes the corresponding class label. The likelihood function associated with these n samples is defined a s ∏_i_ Prob(y_i_∣x_i_). The negative log-likelihood function is called the (empirical) logistic loss, and the average logistic loss is defined as:

$$\text{g}\left(\text{w},\text{c}\right)=-1/\text{n}\log\text{Prob}\left({\text{y}}_{\text{i}}|{\text{x}}_{\text{i}}\right)=1/\text{n}\log\left(1+\exp\left(-{\text{y}}_{\text{i}}\left({\text{w}}^{\text{T}}{\text{x}}_{\text{i}}+\text{c}\right)\right)\right)$$

which is a smooth and convex function. We can determine w and c by minimizing the average logistic loss as follows: min_(w,c)_ g(w,c), which is a smooth convex optimization problem. For high-dimensional data directly solving the logistic regression problem may lead to overfitting. A standard technique to prevent overfitting is regularization. The use of the L_1_ norm regularization leads to the L_1_ regularized logistic regression: min_(w,c)_ g(w,c) + λ||w||_1_, where λ > 0 is a regularization parameter. It is well known that the use of the L_1_ regularization leads to a sparse model, i.e., many of the entries of w are zero, thus achieving feature selection [11]. The resulting optimization problem is convex and non-smooth. In this study, the SLEP (Sparse Learning with Efficient Projections) package that we recently developed is used for solving sparse logistic regression [16].

One major challenge in the use of sparse logistic regression especially for small sample size problems is the estimation of the right amount of regularization (the value of λ), which determines the number of features selected. When λ = 0 all features are likely to be included in the model. As λ > 0 increases, the number of features selected decreases. In this paper, we employed stability selection, a method recently proposed to address the problem of proper regularization using subsampling/bootstrapping [13]. We used bootstrapping in our experiments. The key to stability selection is to perturb the data (e.g. by subsampling or bootstrapping) many times and choose features that occur in a large fraction (determined by a parameter τ described below) of the resulting selection sets. Thus, choosing the right value of the regularization parameter λ becomes much less critical using the stability selection approach, and we have a better chance of selecting truly relevant features. The key steps of stability selection include:

Draw a bootstrap sample B_t_ of size n.

For a given value of the regularization parameter λ (>0), run the sparse logistic regression algorithm on B_t_ to get the optimal solution w^λ^. Denote ${\text{S}}^{\lambda }\left({\text{B}}_{\text{t}}\right)=\left\{\text{j}:{\text{w}}_{\text{j}}^{\lambda }\neq 0\right\}$ as the set of features selected by sparse logistic regression.

Repeat the above two steps N times (t = 1, 2, …, N) and compute the relative selection frequencies: $\prod_{\text{j}}^{\lambda }=\sum_{\text{t}}\text{I}\left(\text{j}\in {\text{S}}^{\lambda }\left({\text{B}}_{\text{t}}\right)\right)/\text{N},\text{j}=1,2,\ldots ,\text{p}$ where I(·) is the indicator function defined as follows: I(g) = 1 if g is true and I(g) = 0 otherwise. That is, $\prod_{\text{j}}^{\lambda }$ is defined as the fraction of bootstrap experiments for which the j-th feature is selected.

Repeat the above procedure for a sequence of M regularization parameters Λ = {λ_1_, λ_1_, .., λ_M_}.

Stability selection outputs the following feature set: ${\text{S}}^{\text{stable}}=\left\{\text{j}:\max_{\lambda \in \Lambda }\prod_{\text{j}}^{\lambda }\geq \tau \right\}$, where τ > 0 is a given threshold value, i.e., a feature is finally selected if, for at least one value of λ, the fraction of bootstra p experiments for which the feature is selected exceeds the threshold τ. In the following, we call $\max_{\lambda \in \Lambda }\prod_{\text{j}}^{\lambda }$ the *stability score* of the j-th feature.

In our experiments, we set N = 1,000, Λ = {i*0.005, i = 1,2,…,60} (M = 60), and τ = 0.5. Our experimental results showed that the classification was not sensitive to τ. Stability selection outlined above is appealing in that it has strong theoretical guarantees. Specifically, it has been shown that subsampling/bootstrapping in conjunction with L_1_-regularized estimation requires much weaker assumptions on the data for asymptotically consistent feature selection than what is needed for the traditional L_1_-regularized scheme [13]. Subsampling/bootstrapping is commonly used for asymptotic statistical inference in terms of standard errors, confidence intervals and statistical testing; one of the distinguishing features of stability selection lies in the marriage of subsampling/bootstrapping and high-dimensional feature selection algorithms which yields finite sample familywise error control and dramatically improves feature selection [13].

We compare sparse logistic regression with stability selection to t-test, which ranks features by calculating a ratio between the difference of two class means and the variability of the two classes [10]. With the selected features (either by *t*-test or sparse logistic regression with stability selection), we apply support vector machines (SVM) to build the classifier [9]. We evaluate the prediction performance of different algorithms in terms of the leave-one-out AUC score.

## Results

### Baseline characteristics

The baseline information of the 319 MCI subjects by diagnostic group (e.g., MCI Converters and MCI Non-converters) is summarized in Table 1. There are no significant between-group differences in age (p = 0.6150) or years of education (p = 0.7093) between the two groups. Both ADAS-cog total 11, which is the 70 point total excluding Q4 (Delayed Word Recall) and Q14 (Number Cancellation), and ADAS-cog total 13, the 85 point total including Q4 and Q14, are significantly higher for MCI Converters than for MCI Non-Converters (p < 0.001); 4 ADAS-cog subscores, including Word Recall (Q1), Delayed Word Recall (Q4), Orientation (Q7), and Word Recognition (Q8), are much higher for MCI Converters (p < 0.001). In addition, between-group differences that represented significantly greater baseline impairment for MCI Converters were noted for MMSE, CDR -SB, LDEL, LIMM, TRAA, TRAB, and FAQ. Finally, MCI Converters were more likely to carry 1 or 2 APOE4 alleles than MCI Non-converters.

**Table 1:** Sample characteristics

	**Non-converter**	**Converter**	**P-Value**
Number of subjects	177 (114/64)	142 (87/55)	
Age	74.90 ± 7.39	74.49 ± 6.94	0.6150
Years of education	15.65 ± 3.06	15.77 ± 2.90	0.7093
MMSE	27.38 ± 1.75	26.62 ± 1.71	<0.001
CDR-SB	1.37 ± 0.75	1.83 ± 0.93	<0.001
ADAS-cog total 11	9.99 ± 4.16	13.09 ± 4.13	<0.001
ADAS-cog total 13	16.06 ± 6.28	21.12 ± 5.79	<0.001
ADAS-cog subscore Q1 Word Recall	4.07 ± 1.36	5.07 ± 1.23	<0.001
ADAS-cog subscore Q2 Commands	0.14 ± 0.47	0.19 ± 0.44	0.3437
ADAS-cog subscore Q3	0.51 ± 0.54	0.56 ± 0.59	0.3931
ADAS-cog subscore Q4 Delayed Word	5.36 ± 2.33	7.12 ± 1.94	<0.001
ADAS-cog subscore Q5 Naming	0.28 ± 0.52	0.22 ± 0.45	0.2380
ADAS-cog subscore Q6 Ideational	0.14 ± 0.40	0.15 ± 0.45	0.7760
ADAS-cog subscore Q7 Orientation	0.39 ± 0.72	0.93 ± 1.10	<0.001
ADAS-cog subscore Q8 Word	4.05 ± 2.68	5.33 ± 2.52	<0.001
ADAS-cog subscore Q9 Recall	0.06 ± 0.38	0.06 ± 0.26	0.9965
ADAS-cog subscore Q10 Spoken	0.05 ± 0.22	0.13 ± 0.46	0.0517
ADAS-cog subscore Q11 Word Finding	0.24 ± 0.57	0.35 ± 0.63	0.0943
ADAS-cog subscore Q12	0.07 ± 0.33	0.09 ± 0.33	0.5268
ADAS-cog subscore Q14 Number	0.78 ± 0.92	1.08 ± 1.09	0.0101
FAQ: Activities of Daily Living	2.41 ± 3.61	5.37 ± 4.70	<0.001
LDEL: Logical Memory delayed	4.59 ± 2.64	2.81 ± 2.32	<0.001
LIMM: Logical Memory immediate	7.77 ± 3.03	6.46 ± 2.95	<0.001
APOE (0 allele/1 allele/2 alleles)	103/59/15	48/71/23	<0.001
TRAA: Trail Making Test: Part A	40.0 ± 15.5	48.1 ± 25.2	<0.001
TRAA: Trail Making Test: Part B	114.7 ± 64.8	144.0 ± 75.2	<0.001

### Pattern classification using baseline measurements

The leave-one-out AUC scores of various baseline measurements and their combinations (without feature selection) are reported in Table 2. Note that the leave-one-out AUC score may be significantly lower than 0.5. Age (AUC = 0.5123), years of education (AUC = 0.5090), the combination of 18 lab tests (AUC = 0.5348), and APOE genotyping (AUC = 0.5473) perform poorly for the discrimination of MCI Non-converters and MCI Converters. MMSE achieves an AUC score of 0.5916 and CDR-SB achieves an AUC score of 0.6064. The combination of 13 ADAS-cog subscores Q1-Q14 (AUC = 0.7598) achieves a higher AUC score than each of the 13 ADAS-cog subscores and both ADAS-cog total 11 (AUC = 0.7024) and ADAS-cog total 13 (AUC = 0.7248). Among the 13 ADAS-cog subscores, ADAS-cog subscore Q1 (AUC = 0.6830) and ADAS-cog subscore Q4 (AUC = 0.6842) achieve the best performance. The combination of ADAS total 13, ADAS subscores, MMSE, and CDR-SB perform better than individual scores. A combination of 237 MRI features (see the list of MRI features in the supplemental document) achieves an AUC score of 0.7214, FAQ achieves an AUC score of 0.6874, and TRAB (AUC = 0.6187) performs slightly better than TRAA (AUC = 0.5944).

**Table 2:** Prediction performance of various baseline measurements and their combinations in terms of the AUC Score

	**AUC score**
ADAS-cog total 11	0.7024
ADAS-cog total 13	0.7248
ADAS-cog subscore Q1 Word Recall	0.6830
ADAS-cog subscore Q2 Commands	0.1581
ADAS-cog subscore Q3 Construction	0.4899
ADAS-cog subscore Q4 Delayed Word	0.6842
ADAS-cog subscore Q5 Naming	0.3202
ADAS-cog subscore Q6 Ideational	0.5142
ADAS-cog subscore Q7 Orientation	0.4836
ADAS-cog subscore Q8 Word	0.6062
ADAS-cog subscore Q9 Recall	0.2581
ADAS-cog subscore Q10 Spoken	0.3914
ADAS-cog subscore Q11 Word Finding	0.5436
ADAS-cog subscore Q12 Comprehension	0.2756
ADAS-cog subscore Q14 Number Cancellation	0.4451
ADAS-cog subscore Q1-Q14	0.7598
Age	0.5123
Years of Education	0.5090
MMSE Score	0.5916
CDR-SB	0.6064
ADAS total 13 + ADAS subscores	0.7561
ADAS total 13 + ADAS subscores + MMSE + CDR-SB	0.7674
FAQ	0.6874
LDEL: Logical Memory delayed	0.6573
LIMM: Logical Memory immediate	0.6136
MRI (237)	0.7214
Lab tests (18)	0.5348
APOE genotyping	0.5473
TRAA: Trail Making Test: Part A	0.5944
TRAA: Trail Making Test: Part B	0.6187

The numbers in the second column are the leave-one-out AUC score which may be significantly lower than 0.5. The number in the parenthesis denotes the number of measurements involved.

### Data integration and biosignature selection

**Figure 2:**
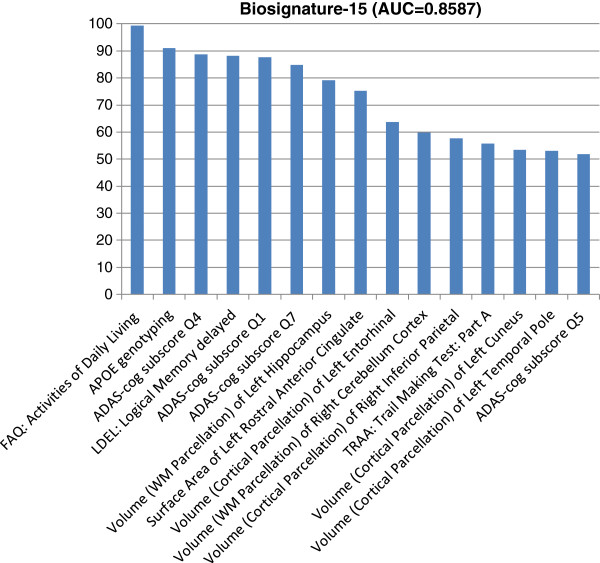
**The top 15 features (included in Biosignature-15) identified by sparse logistic regression with stability selection.** The vertical axis is the stability score multiplied by 100 (between 0 and 100) and indicates the importance of the features. WM indicates White Matter.

**Figure 3:**
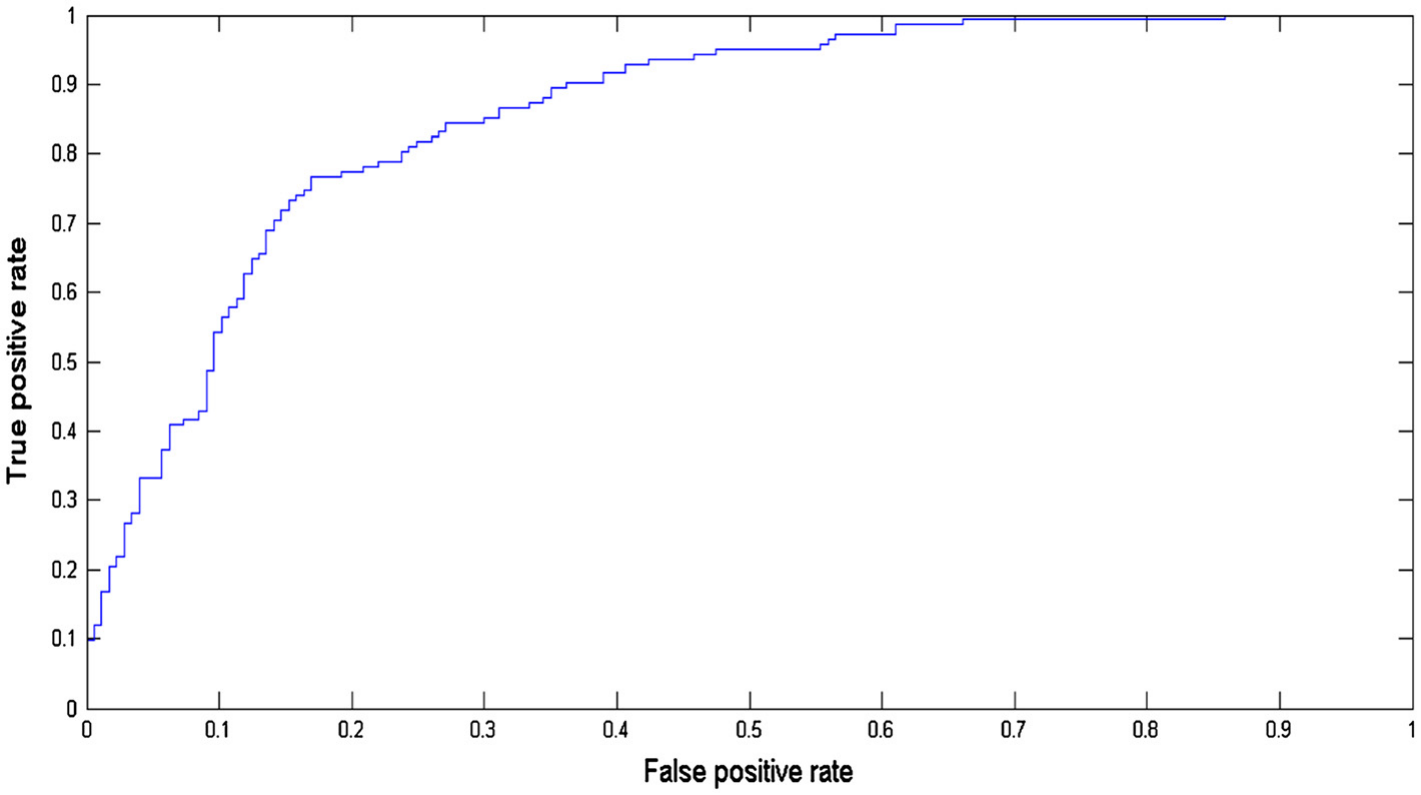
**The AUC Curve of *Biomarkers-15 *by sparse logistic regression with stability selection**.

Next, we study the integration of various baseline measurements for predicting the conversion from MCI to probable AD and identify an optimal biosignature for the prediction. We examine two feature selection algorithms, including univariate feature ranking based on the *t*-test and sparse logistic regression with stability selection. Univariate feature ranking achieves an AUC score of 0.7935 by using the top 15 features, while sparse logistic regression with stability selection achieves an AUC score of 0.8587 by using a total of 15 features. The top 15 features identified by the stability selection (listed in Figure 2) include FAQ: Activities of Daily Living Score, APOE genotyping, ADAS-cog subscore Q4 (Delayed Word Recall), Logical Memory delayed, ADAS-cog subscore Q1 (Word Recall), ADAS-cog subscore Q7 (Orientation), Volume (White Matter Parcellation) of Left Hippocampus, Surface Area of Left Rostral Anterior Cingulate, Volume (Cortical Parcellation) of Left Entorhinal, Volume (White Matter Parcellation) of Right Cerebellum Cortex, Volume (Cortical Parcellation) of Right Inferior Parietal, TRAA: Trail Making Test: Part A, Volume (Cortical Parcellation) of Left Cuneus, Volume (Cortical Parcellation) of Left Temporal Pole, ADAS-cog subscore Q5 (Naming). For convenience we call this set of 15 features “*Biosignature-15*” in the following discussions. The corresponding AUC curve is shown in Figure 3.

**Figure 4:**
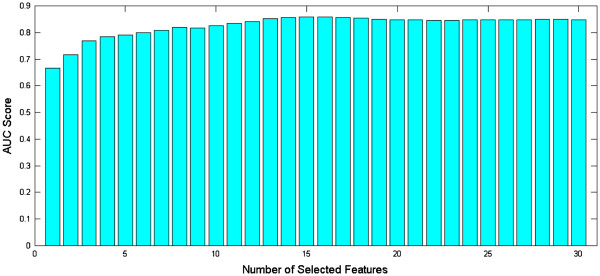
The change of the AUC score when the number of selected features varies.

To demonstrate the stability of sparse logistic regression with stability selection, we conduct the classification using the top T features for T = 1, 2, …, 30, and the results are shown in Figure 4. The performance in terms of the AUC score is not sensitive to the number of features selected. The AUC score stabilizes after the top 13–15 features are included; including any additional features will not further improve the performance. Our results demonstrate the effectiveness of stability selection. To examine the added benefit of integrating MRI features with various demographic, genetic, and cognitive measurements, we apply sparse logistic regression with stability selection on MRI features alone. The top 10 MRI features identified by stability selection are listed in Table 3 (left column), and the AUC score is 0.7877. Table 2 shows that the combination of 237 MRI features achieves an AUC score of 0.7214. Sparse logistic regression with stability selection on the MRI features significantly improves the performance; the AUC score improves from 0.7214 to 0.7877 (p-value < 0.05). In addition, we apply sparse logistic regression with stability selection on the combination of different demographic, genetic, and cognitive measurements excluding MRI features. The top 10 demographic, genetic, and cognitive measurements identified by stability selection are listed in Table 3 (right column), and the AUC score is 0.8111. The AUC of Biosignature-15 is statistically greater than the AUCs of the top MRI features and the top 10 demographic, genetic, and cognitive measurements (p-value < 0.05). Most (top) items in Figure 2 and Table 3 match; the differences are possibly due to the correlation among different measurements, especially the correlation between the MRI features and various demographic, genetic, and cognitive measurements.

**Table 3:** The top 10 MRI features (left column) and demographic, genetic, and cognitive measurements (right column) identified by sparse logistic regression with stability selection are ordered in decreasing order of stability scores

**MRI**	**Demographic, genetic, and cognitive**
Volume (WM Parcellation) of Left Hippocampus	FAQ: Activities of Daily Living
Volume (Cortical Parcellation) of Left Entorhinal	APOE genotyping
Surface Area of Left Rostral Anterior Cingulate	LDEL: Logical Memory delayed
Volume (Cortical Parcellation) of Right Inferior Parietal	ADAS-cog subscore 4
Cortical Thickness Average of Left Isthmus Cingulate	ADAS-cog subscore 1
Volume (Cortical Parcellation) of Left Cuneus	ADAS-cog subscore 7
Volume (WM Parcellation) of Right Amygdala	ADAS-cog subscore 5
Cortical Thickness Average of Right Entorhinal	TRAA: Trail Making Test: Part A
Volume (WM Parcellation) of Left Amygdala	ADAS-cog subscore 10
Cortical Thickness Average of Left ParsOrbitalis	Years of Education

*WM* indicates White Matter.

### Integration of CSF and other measurements

We extracted a subset of 160 MCI subjects (74 MCI Converts, 86 MCI Non-converters) with a complete set of CSF measurements and MRI scans. We first test the ability of individual CSF biomarkers including t-tau, Aβ42, p-tau, and two ratios (t-tau/Aβ42 and p-tau/Aβ42) for predicting the conversion from MCI to probable AD, and the results are summarized in Table 4. Similar to a previous study [7], we evaluated the performance of combing individual CSF biomarkers with the biosignatures included from the larger data set excluding CSF measurements called Biosignature-15 (see Figure 2). The results in Table 4 showed that (1) the CSF biomarkers do not perform as well as Biosignature-15; and (2) the inclusion of the CSF biomarkers does not improve the performance of Biosignature-15, although the difference is not statistically significant. Note that the AUC for Biosignature-15 reported in Table 4 included only the subset of 160 subjects with CSF measurements.

**Table 4:** **Prediction performance of various baseline CSF measurements and the combinations of CSF measurements and *Biomarkers-15 *in terms of the AUC score **

	**AUC score**
CSF t-tau	0.616
CSF Aβ_42_	0.612
CSF p-tau	0.628
CSF t-tau/Aβ_42_	0.631
CSF p-tau/Aβ_42_	0.634
*Biomarkers-15 *	0.830
*Biomarkers-15 * + CSF t-tau	0.826
*Biomarkers-15* + CSF Aβ_42_	0.827
*Biomarkers-15 * + CSF p-tau	0.827
*Biomarkers-15 * + CSF t-tau/Aβ_42_	0.826
*Biomarkers-15 * + CSF p-tau/Aβ_42_	0.827

Note that the AUC score for Biosignature-15 reported in this table included only the subset of 160 subjects with CSF measurements.

## Discussion

These results demonstrate the effectiveness of sparse logistic regression with stability selection for (1) integrating various baseline data from ADNI (MRI, demographic, genetic and cognitive measures) for predicting the conversion from MCI to probable AD; and (2) identifying a small set of strong predictors.

Many of the selected features in Biosignature-15 have been identified to be important in characterizing AD. Biosignature-15 includes 3 ADAS-cog subscores (Q4, Q1, Q7) in the top 6. These three subscores contribute the largest weights to the ensemble tree-based predictive model (Random Forest) in [17] and are primarily tests of memory, a key cognitive domain affected early by the disease. Specifically, Q1 and Q4 are memory tests, which have face validity; Q7 is orientation but involves memory to recall the date, time of day, and place.

Most of the MRI features in Biosignature-15 are volumes known to be reduced in AD. The hippocampus and entorhinal cortex have long been known as the first areas to be affected in Alzheimer’s Disease, both on histology and via gross morphological changes visible on imaging [18-23]. The entorhinal cortex is located in the medial temporal lobe and functions as a hub in a widespread network for memory and navigation. The hippocampus is also located in the medial temporal lobe and plays important roles in memory, both for registration and recall and spatial navigation. Changes in the temporal region have been shown to be a good predictor of the progression of AD [24,25]. Other studies have also detected a surprising correlation between cerebellar atrophy and AD, as have we. It was demonstrated in [26,27] that the atrophy of the cerebellum, a brain region not associated with the cortical pathology of AD or typically thought to have a role in cognition and generally believed to be involved only late in AD, was found to be significantly correlated with clinical severity of the disease. One study [28] suggested that metabolism in areas such as cerebellum was correlated with deficits in neuropsychological function. Finally, it has been shown previously that the rostral anterior cingulate is affected in AD [5,29]. The anterior cingulate cortex is cytoarchitectonically and functionally divided into parts; the rostral division has connections to limbic and paralimbic structures including the amygdala and hippocampus. The atrophy of the caudal portion of the anterior cingulate was shown to be predictive of conversion to AD in memory impaired subjects, suggesting that this structure might be affected relatively early in the course of the disease [30,31]. All four cingulate regions were shown to be significantly smaller in AD cases compared with controls; the atrophy in th e posterior cingulate region was significantly greater than that in other cingulate regions [29]. Several previous studies [4,25,32] also achieve good prediction performance; however, in all these studies, the classes (MCI Converts and MCI Non-Converts) were stratified by age, and thus age was also predictive. In several other studies, as in ours, age does not stratify the two classes, and thus is not a relevant predictor. Querbes et al. (2009) developed a normalized thickness index which was computed using the subset of regions (right medial temporal, left lateral temporal, right posterior cingulate) and achieved an AUC score of 0.76 [33]. There were 122 MCI subjects used in this study including 50 MCI Non-converters and 72 MCI Converters (the conversion was considered over the course of a 2-year time period). In their study, age, years of education, MMSE, and Trail Making test B achieved an AUC score of 0.52, 0.53, 0.64, and 0.72, respectively. Misra et al. (2009) used MRI scans to predict the short-term conversion from MCI to AD and achieved an AUC score of 0.77 [34]. There were 103 MCI subjects used in that study including 76 MCI Non-converters and 27 MCI Converters. In comparison, we achieve a higher AUC score (0.8587) with a larger sample size (319 MCI subjects) and a larger number of baseline measurements.

The combination of demographic, genetic, and cognitive measurements outperforms MRI alone for predicting the MCI to AD conversion. These demographic, genetic, and cognitive measurements can potentially be used to pre-screen a large number of participants for large-scale AD studies. In addition, stability selection provides a small subset of candidate demographic, genetic, and cognitive measurements (see Table 3) for effective and efficient screens. In a recent study [17], an ensemble tree- based predictive model (Random Forest) was built to predict MCI Converters within 1 year. Their results show that the addition of MRI features to the cognitive markers did not achieve performance gain. However, our results show that the integration of demographic, genetic, and cognitive measurements and MRI features using sparse logistic regression with stability selection achieves a much higher AUC score (AUC = 0.8587) than MRI markers alone. The result demonstrates the benefit of integrating MRI features with various demographic, genetic, and cognitive measurements for the prediction. In our study, we assume that various types of baseline data (MRI, demographic, genetic and cognitive measures) are available in deriving Biosignature-15. However, this may not be case in clinical practice.

The results in Table 4 showed that the CSF biomarkers are not very effective for the MCI-to-AD prediction. Shaw et al. (2009) showed that CSF measurements are the most informative markers for distinguishing AD patients from normal controls and the differences between MCI Converters and MCI Non-Converters are significant [6]. However, their analysis is based on a total of 37 MCI subjects. It is mentioned in the paper: “Because of the small numbers of subjects, it is important to be cautious about drawing any definitive conclusions from these subjects.” A recent study [7] conducted by the same group showed that MRI and CSF achieved the best AUC score of 0.734, the CSF biomarkers performed slightly worse than MRI features, and the combination of MRI and CSF achieved a lower AUC score than MRI. In [35], mixture modeling approaches were used to apply the CSF measurements in the diagnosis of AD. The proposed approach showed 100% sensitivity in 57 patients with MCI who were clinically progressing towards dementia over the course of a 5-year time period. However, no specificity result was reported. We find that while in the current ADNI cohort, all MCI Converters show an aberrant CSF signature (defined as high p-tau i.e. >23 pg/mL and low Aβ_42_ i.e. <192 pg/mL), such a CSF signature is also present in many MCI Non-converters. It has been surmised that the subset with the CSF signature will likely convert to AD in the future. However, ADNI is an on-going study, and based on the data currently available, CSF markers do not show enough specificity to discriminate between MCI to AD Converters and Non-converters.

Our findings are consistent with several recent reports in the literature. In [36], no association between MMSE change and change in levels of CSF biomarkers was reported, whereas brain atrophy was predictive of MMSE change. Vemuri et al. (2009) investigated the relationship between baseline MRI and CSF biomarkers and subsequent change in cognitive and functional abilities, which were modeled as average CDR–SB and MMSE scores over a 2-year period [37]. Their results showed that MRI biomarkers were better predictors of subsequent cognitive/functional change than CSF biomarkers. In a recent study [8], it was shown that baseline MRI morphometry was more related to clinical change as indexed by CDR-SB than were CSF biomarkers. These studies suggest a stronger association between brain atrophy measured by MRI and progression of clinical symptoms measured by CDR–SB and/or MMSE than between CSF levels and progression of clinical symptoms. The results presented in this paper are consistent with these observations.

## Conclusions

In this paper we have demonstrated the application of sparse logistic regression and stability selection for integrating various baseline ADNI data (MRI, CSF, demographic, genetic, and cognitive measures) for predicting the conversion from MCI to probable AD and identifying a small subset of biosignatures for the prediction. Sparse logistic regression with stability selection combines the strengths of two approaches well-known in the literature to yield a robust set of biosignatures, called *Biosignature-15*. We further show that sparse logistic regression with stability selection achieves very good predictive performance, with an AUC of 0.8587, which is higher than previous known results using data that, similar to ours, are not age-stratified. It is important to note that ADNI is single homogeneous sample of highly educated and motivated volunteers. Additional studies are required to test the generalization ability of *Biosignature-15*. In addition, further analysis is needed to determine whether the combination of various baseline measurements can predict the time-to-conversion. Finally, we plan to examine the influence of other common comorbidities on the prediction model such as cardiovascular risk factors disease and depression, family history of dementia, prior head trauma etc.

## Competing interests

The authors declare that they have no competing interests.

## Authors’ contributions

All authors analyzed the results and wrote the manuscript. JY and VN conceived the project and designed the methodology. JY implemented the programs and drafted the manuscript. All authors have read and approved the final manuscript. Data used in preparation of this article were obtained from the ADNI database (adni.loni.ucla.edu). As such, the investigators within the ADNI contributed to the design and implementation of ADNI and/or provided data but did not participate in analysis or writing of this report. A complete listing of ADNI investigators can be found at: adni.loni.ucla.edu/wp-content/uploads/how_to_apply/ADNI_Acknowledgement_List.pdf.

## Pre-publication history

The pre-publication history for this paper can be accessed here:

http://www.biomedcentral.com/1471-2377/12/46/prepub

## Supplementary Material

Additional file 1 MRI feature names. Click here for file
